# Contribution of Singular Spectral Analysis to Forecasting and Anomalies Detection of Indoors Air Quality

**DOI:** 10.3390/s22083054

**Published:** 2022-04-15

**Authors:** Felipe Espinosa, Ana B. Bartolomé, Pablo Villoria Hernández, M. C. Rodriguez-Sanchez

**Affiliations:** 1 Electronics Department, University of Alcala, E-28801 Alcalá de Henares, Spain; felipe.espinosa@uah.es (F.E.); belen.bartolome@edu.uah.es (A.B.B.); 2 Electronics Technology Department, University Rey Juan Carlos, E-28933 Móstoles, Spain; pablo.villoria@getronics.com

**Keywords:** air quality monitoring, Singular Spectral Analysis, time series modelling, treepartition modelling, forecasting, anomalies detection

## Abstract

The high impact of air quality on environmental and human health justifies the increasing research activity regarding its measurement, modelling, forecasting and anomaly detection. Raw data offered by sensors usually makes the mentioned time series disciplines difficult. This is why the application of techniques to improve time series processing is a challenge. In this work, Singular Spectral Analysis (SSA) is applied to air quality analysis from real recorded data as part of the Help Responder research project. Authors evaluate the benefits of working with SSA processed data instead of raw data for modelling and estimation of the resulting time series. However, what is more relevant is the proposal to detect indoor air quality anomalies based on the analysis of the time derivative SSA signal when the time derivative of the noisy original data is useless. A dual methodology, evaluating level and dynamics of the SSA signal variation, contributes to identifying risk situations derived from air quality degradation.

## 1. Introduction

Air quality, indoors and outdoors, has a direct impact on the environment and, fundamentally, on the health and comfort of citizens [1]. Improving air quality is therefore a challenge for both public administrations and private entities. The tendency of populations to concentrate in large cities has increased the interest in this topic, hence the continuous references in publications related to Smart Cities [2,3,4,5,6].

To assess air pollution, different pollutants are considered: carbon monoxide and dioxide, ozone, nitrogen dioxide and sulphur dioxide. Although these are not detectable with the naked eye, there are commercial devices that include the necessary algorithm to report standardised air quality indices (AQ Index) [7,8,9]. Besides, there are visible air pollutants in the form of solid particles emitted to the atmosphere, particulate matter PM10 and PM2.5 [10].

The starting point for monitoring air quality is to have sensor devices [11], generally forming wireless sensor network nodes supported by IoT technology [5,12,13]. Subsequent data processing is essential, both to have models that anticipate the evolution of this variable, and to alert researchers to anomalous variations detected in its recording.

The record provided by air quality sensors usually incorporates significant noise components. Part of this noise is due to the nature of the variable itself, conditioned by climatological phenomena (temperature, humidity, atmospheric pressure), and part to the characteristic noise of the sensor itself. Therefore, the processing of the original signal is essential to facilitate its subsequent analysis, modelling and prediction. In the case of univariate time series, techniques such as autocorrelation and periodogram are traditionally used to reduce the noise components inherent to the recording of time series; however, these techniques have significant limitations in defining the number of regressors in the modelling of the series [14,15].

**Figure 1:**
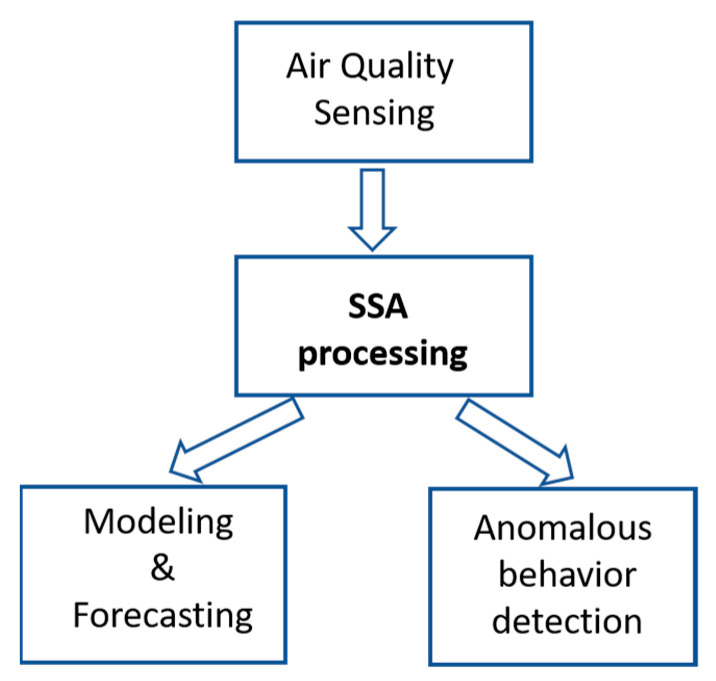
Contribution scheme of SSA to air quality forecasting and anomalous detection.

The contribution of this work focuses on the application of Singular Spectral Analysis (SSA) to the processing of data from air quality sensors. From the principal components of the signal, a double objective is proposed: to obtain a model that allows predicting its evolution with a defined horizon and to analyse the temporal evolution changes in order to detect risk situations due to the spontaneous or forced introduction of pollutants. Figure 1 shows a schematic presentation of this contribution.

The authors’ contribution to smart node Yongzhi sensing can be extended to a spatially distributed wireless sensing network as described in [13,16] in the Smart City context.

## 2. SSA Fundamentals

Singular Spectrum Analysis (SSA) is a powerful technique in time series analysis with a diverse application field. The first publications date from 1986, but theoretical and methodology aspects can also be found in several recent references [16,17,18]; this is why a mathematical review is included in this chapter.

The aim of SSA is to make a decomposition of the original series into the sum of a small number of independent and interpretable components and a structureless noise. This way, slowly varying trends, seasonality components or cycles with small and large periods can be isolated without needing to know the model of the original signal.

We consider a real-valued nonzero time series of length *N*, $X_{N}=\left(x_{1},\;\ldots \;x_{N}\right)$. The main purpose of SSA is to decompose the original series into a sum of components (identified as dominant/principal components) and noise. This is followed by a reconstruction the original series.

Given a window length L (1<L<N), we construct the *L*-lagged vectors:

(1) $$X_{i}={\left(x_{i},\;\ldots \;x_{i+L-1}\right)}^{T},\;i=1,\;2,\;\ldots ,\;K=N-L+1$$

and compose these vectors into the X matrix:

(2) $$X={\left(x_{i+j-1}\right)}_{i,j=1}^{L,K}=\left[X_{1}:\ldots :X_{K}\right]$$

It is a Hankel (square and symmetric) matrix with size LxK and is often called `trajectory matrix’. The singular-value decomposition of the matrix $C=XX^{T}$ yields a collection of L eigenvalues $\lambda_{i}$ and eigenvectors $v_{i}$:

(3) $$C=E\Lambda E^{T}$$

where **Λ** is a diagonal matrix containing the singular values ($\sigma_{i}=\sqrt{\lambda_{i}},$) of C and $E=\left[v_{1}\ldots .v_{L}\right]$ is the orthogonal matrix formed with the eigenvectors $v_{i}$.

**Figure 2:**
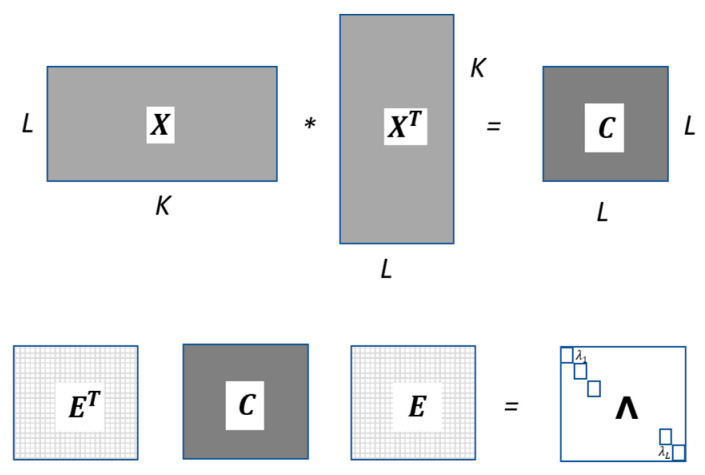
Steps involved in the SSA decomposition stage.

**Figure 3:**
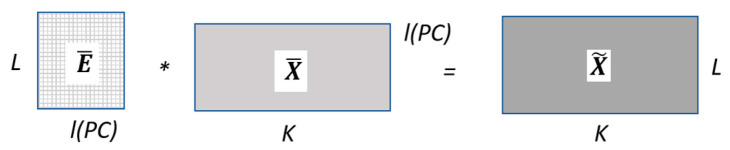
SSA reconstruction stage from the principal components.

Operating with E and C matrices, the diagonal (eigenvalues) **Λ** matrix is obtained as shown in Figure 2. A particular combination of a certain number l<L (l or principal components) of these eigenvectors, determines an l-dimensional subspace in ${\mathbb{R}}^{L}$. The L-dimensional data $X=\left\{X_{1},\;\ldots ,\;X_{K}\right\}$ is then projected onto this l-dimensional subspace $\bar{E}$ to obtain the $\bar{X}$ submatrix (sized Kxl) that allows to reconstruct the Hankel matrix $\tilde{X}$, as symbolically described in Figure 3. The $\tilde{X}$ is considered as an approximation to X, where the parameters L and l (Principal Components—PC) are chosen by the designer for a specific application.

## 3. HelpResponder Project—Case Study: Air Quality Evaluation

There are several alternatives and devices for monitoring air quality. In [19], an MQ135 sensor is used for its wide range for gases detection (NH3, NOx, CO2, etc.). In [8], a low-cost device to measure Indoor Air Quality (IAQ) is used: BME680. The gas sensor BME680 is a digital device capable to measure gas (in a unit of Ohm), humidity (RH), pressure (hPa) and temperature (°C) that complies with ISO16000-29 standard “Test methods for Volatile Organic Compounds (VOC) detectors”. The operating range of BME680 is −40/+85 °C, 0/100% RH and 300/1100 hPa, which suits general conditions. The IAQ is calculated by a smart algorithm using these measurement parameters, developed by Bosch Software Environmental Cluster (BSEC) based on the German Federal Environmental Agency’s guidelines.

The interpretation of computed IAQ is shown in Table 1, where lower IAQ infers better air quality [20]. The combination of both gas and chemoresistive sensors allows the calculation of an Air Quality Index based on the concentration of volatile organic compounds (VOCs), CO and NOx.

The authors belong to the research team involved in the HelpResponder project. The global goal of the project is to provide Emergency Professionals with innovative technology making their emergency activity easier and guaranteeing their own safety as well as that of the people to be evacuated. The final platform deals with the integration of electronics, communication and services that increase the performance of portable equipment and its interaction with Management and Supervision Staff. Among the tasks to be carried out is the detection of level and degradation of indoor air quality.

**Figure 4:**
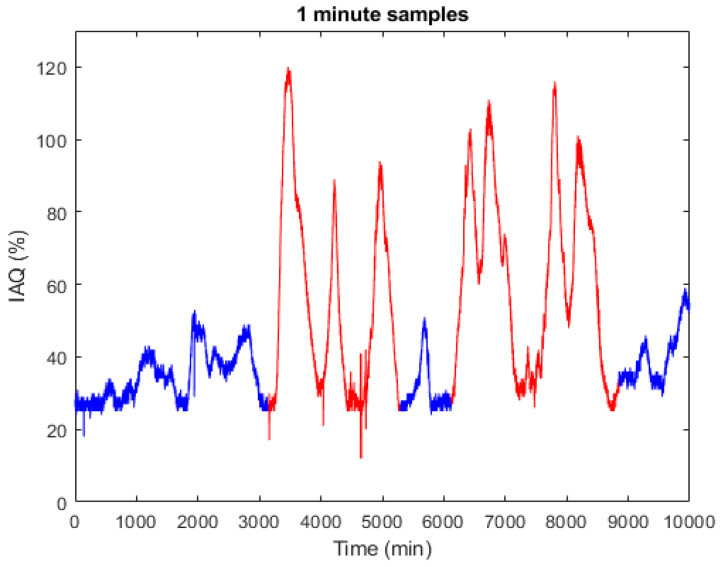
Recorded IAQ data under normal condition and incorporating smoke int the indoor area under test.

In this context, Figure 4 shows an actual record obtained at the Alcorcón Fire Department (Madrid). The test was carried out between 27 October and 3 November 2020; recording IAQ values provided by a BME680 sensor with a 1 min sampling. Over some time intervals, smoke was introduced to force a degradation of the air quality. As can be seen, between minute 3000 and 8800 there are intervals with very bad IAQ levels (see Table 1). The records in blue show results obtained under normal condition and the data in red those obtained under forced disturbance condition.

## 4. SSA Contribution to Air Quality Estimation

**Figure 5:**
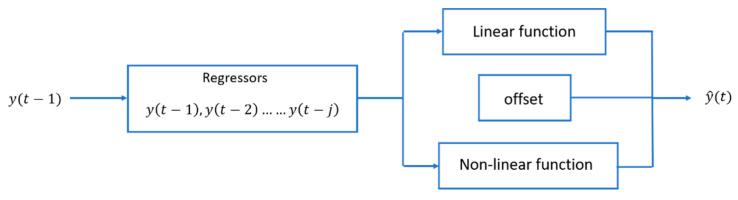
Scheme of non-linear time series modelling.

The next measurement estimation or the variable prediction with a given time horizon requires a model of time series under study. In the case of non-linear time series such as air quality measurements, intelligent different solutions have been recently published: based on shallow and deep-learning predictors [21], neuro-fuzzy network approach [22] or alternatives based on support vector machines [23]. However, to highlight the SSA contribution to this goal, a first approach combining linear and non-linear modelling techniques is discussed, as shown in Figure 5, from a set of j regressors, and adjusting the corresponding offset.

In this work we have used a solution in which the linear component responds to an autoregressive AR model; for the non-linear component we have compared three solutions: a sigmoidal function, another based on the wavelet transform and another based on the treepartition technique. Matlab has specific functions for each (sigmoidnet, wavenet and treepartition) [24,25,26]. The main configuration parameters are the number of regressors (order) and the number of non-linear functions (units), which can be chosen by the user or set automatically.

To evaluate the SSA contribution to air quality modelling, the following methodology is proposed: (a) modelling and prediction of the time series from the original IAQ data provided by the sensor; (b) modelling and prediction of the time series after SSA data processing.

For the first phase of the study, the original 3000 data recorded under normal (smokeless) condition were selected. The first 2000 data were used for identification and the last 1000 for validation [27]. A minimum modelling fit (FIT) of 80% was set as an acceptance criterion, and automatic identification was carried out for the three alternatives previously mentioned. The validation results for an order of 50 are shown in Table 2.

After the obtained results, the treepartition model of 50 order and 31 units was chosen, as it presented the lowest MSE (Mean Squared Error) with the shortest computation load. The relative value t = 1 corresponds to 1.447 s using an HP Pavilion x360 Convertible PC, with an Intel Core i7-7500U processor, 2 cores, 12 GB DDR4 RAM and a NVIDIO GeForce 940 MX graphics card.

**Figure 6:**
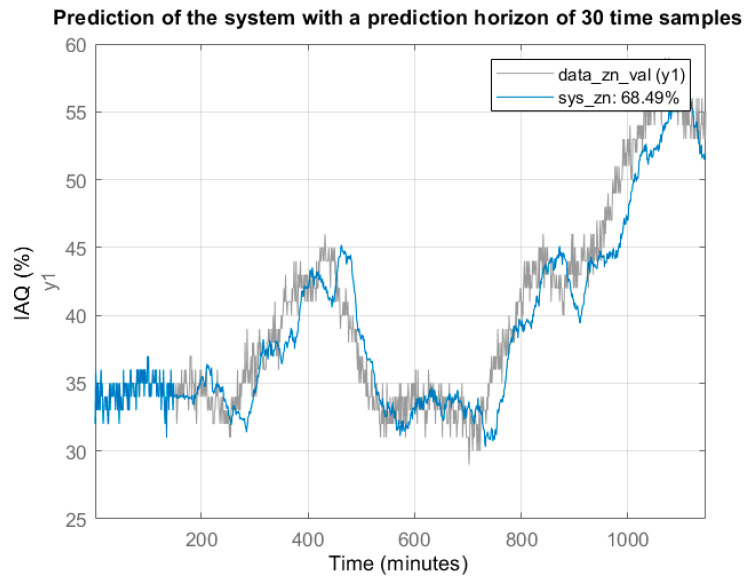
Comparison between original signal (do not included at the identification stage) and estimation with the treepartition model proposed by authors.

Considering this model, an estimation was made with a half-hour horizon (30 samples) and the model results were compared with the recorded results (last 1100 data in Figure 4). In Figure 6, the grey trace represents part of the original signal data_zn_val(y1) and the blue trace is the estimation provided by the model with the indicated time horizon. The percentage of fit is slightly higher than 70% mainly due to the limitations of the model to fit the original signal with significant noise content.

**Figure 7:**
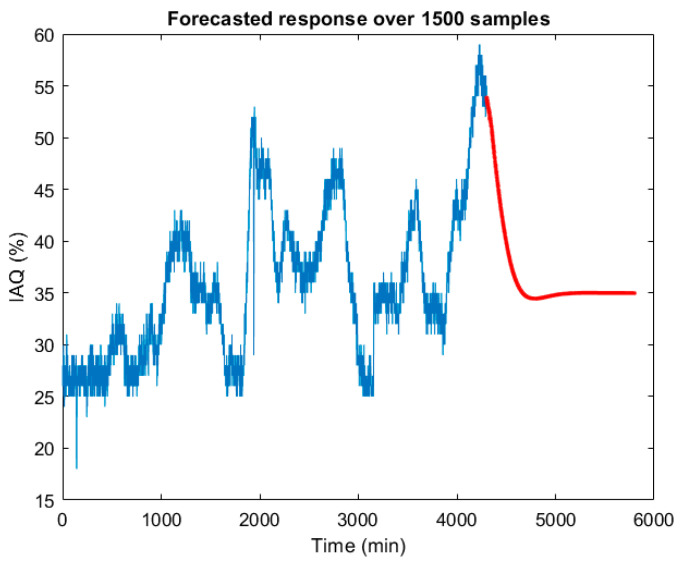
IAQ prediction with a half-hour horizon, from the treepartition time series model.

One of the objectives of time series modelling is to predict the variable under study beyond the last recorded sample. For this purpose, the Matlab forecast function [25,26] was used in this work. Figure 7 shows the IAQ prediction results for half an hour after the last recorded data.

**Figure 8:**
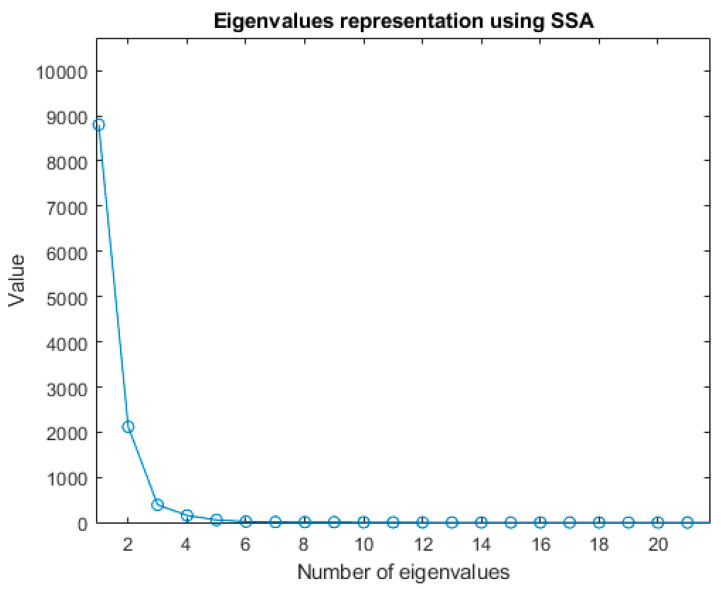
Eigenvalue representation applying SSA to the IAQ time series, being the time window L = 250.

**Figure 9:**
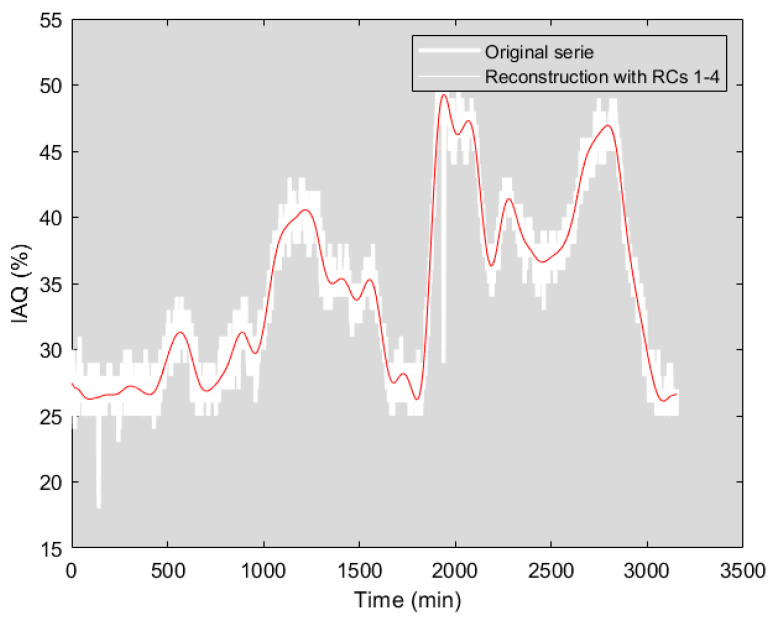
IAQ time series (without disturbances) reconstruction with *L* = 250 and four dominant eigenvalues.

In the following, the SSA contribution to air quality modelling and prediction is evaluated. Taking as reference the works of [17,28], a time window *L* ≈ *N*/12 (250 samples) was chosen, where N is the total of the recorded data for the analysis in the time interval without forced disturbance. The covariance matrix was calculated and the eigenvalues shown in Figure 8 were obtained. The signal reconstruction obtained with *L* = 250 and the dominant eigenvalues (RCs = 4) is shown in Figure 9; the percentage of fit is 80.27%. As indicated in Table 3, increasing the number of principal components (eigenvalues) does not significantly improve the reconstruction percentage of the time series.

**Figure 10:**
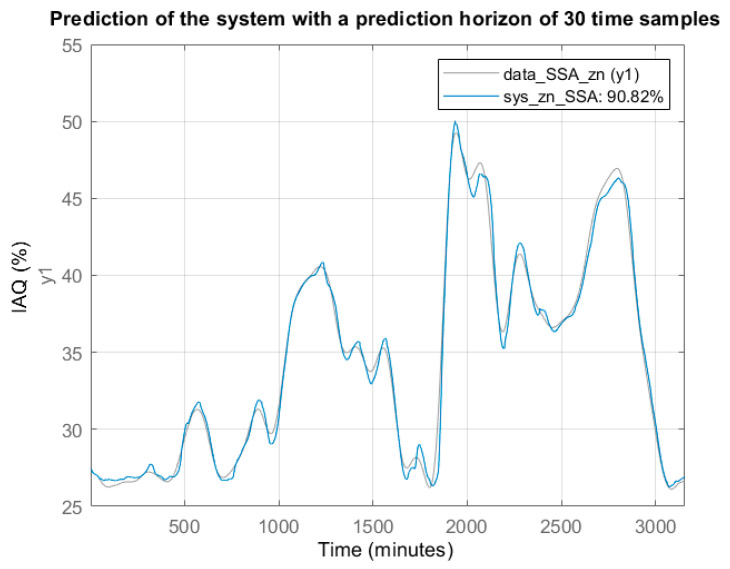
Estimation result using the treepartition model (50 order and 31 units) from the SSA reconstructed signal instead of the original one.

**Figure 11:**
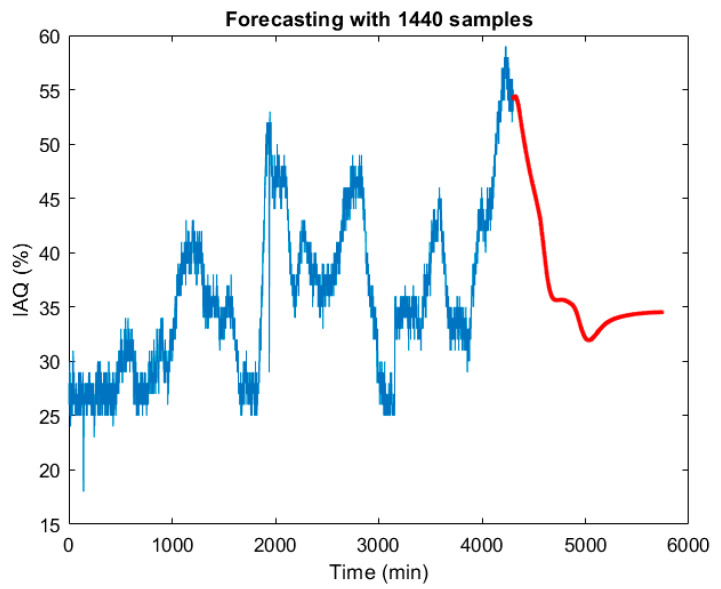
One day IAQ prediction (1440 min) based on the SSA processing of the original measurements.

Using the same nonlinear model structure (treepartition, 50 order and 31 units), but with the SSA processed data, the model validation results are: Fit = 99.12%, MSE = 0.0035 and computation time 0.71 s. Figure 10 shows the graphical modelling results. Compared to the modelling results in Table 2, obtained using the raw data (original signal), the MSE was divided by 433, the Fit was improved by 16.88% and the computation time was reduced by 51%. The same model is applicable to the prediction of IAQ evolution. Figure 11 shows the one-day prediction (1440 samples, with a period of one minute). Compared to the prediction in Figure 6, the one obtained from the SSA processed signal shows a smoother dynamic.

## 5. Air Quality Anomalies Detection

**Figure 12:**
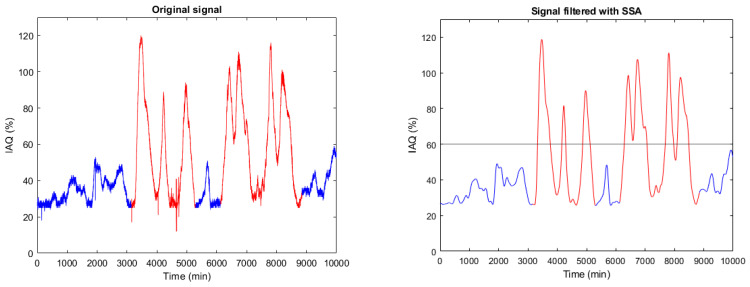
Original (**left**) and SSA processed (**right**) IAQ signal. The blue line belongs to registered data in normal conditions, while the red line is related to forced disturbances in the scenario under test. According to Table 1, 60% is the threshold to consider very bad air quality.

Air quality degradation can have different causes. These can be natural, such as the increase of COx, NOx, Sox or O3 levels, generally with slow dynamics, or forced by emergency situations such as fire, criminal actions with chemical components, etc. In any case, these anomalies result in very bad IAQ levels, above 60 (see Table 1) and fast dynamics. This is the case of the forced disturbances shown in red in Figure 12.

**Figure 13:**
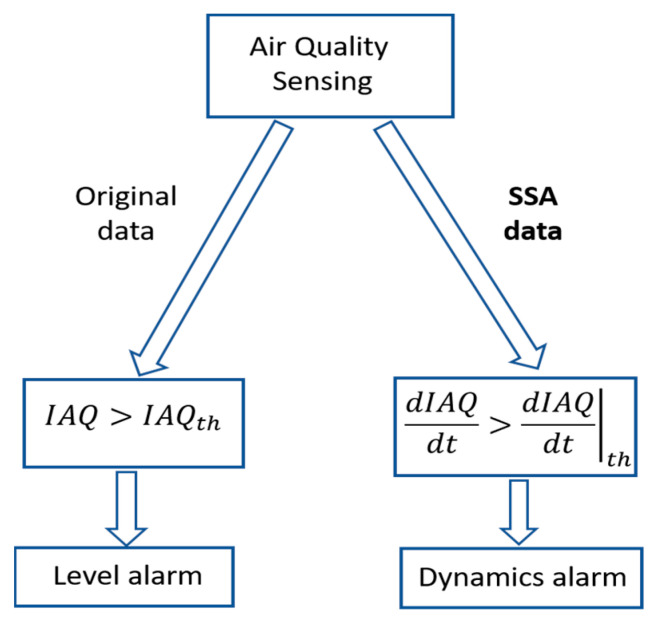
Dual methodology for air quality anomalies detection.

**Figure 14:**
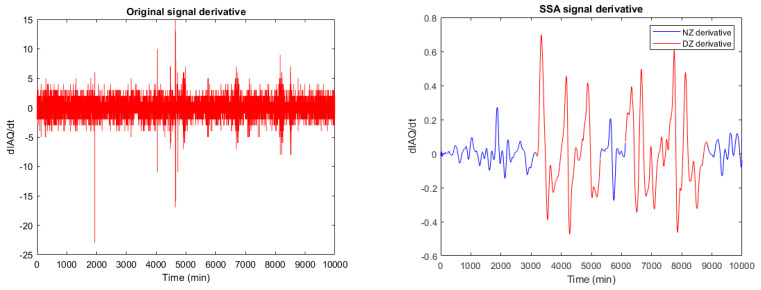
Original (**left**) and SSA processed temporal derivative (dIAQ/dt) signal (**right**).

Knowing the critical consequences of IAQ degradation, the authors propose a dual approach to characterise areas of anomalous behaviour: level and dynamic detection, as shown in Figure 13. A level alarm will be generated when the IAQ value exceeds the threshold $IAQ_{th}$ = 60% (very bad IAQ according to Table 1). In the case of dynamic detection, it is unfeasible to draw conclusions from the time derivative of the original signal (raw data), as shown in Figure 14(left). However, the time derivative of the SSA processed signal (Figure 14(right)) provides relevant information about anomaly detection.

**Figure 15:**
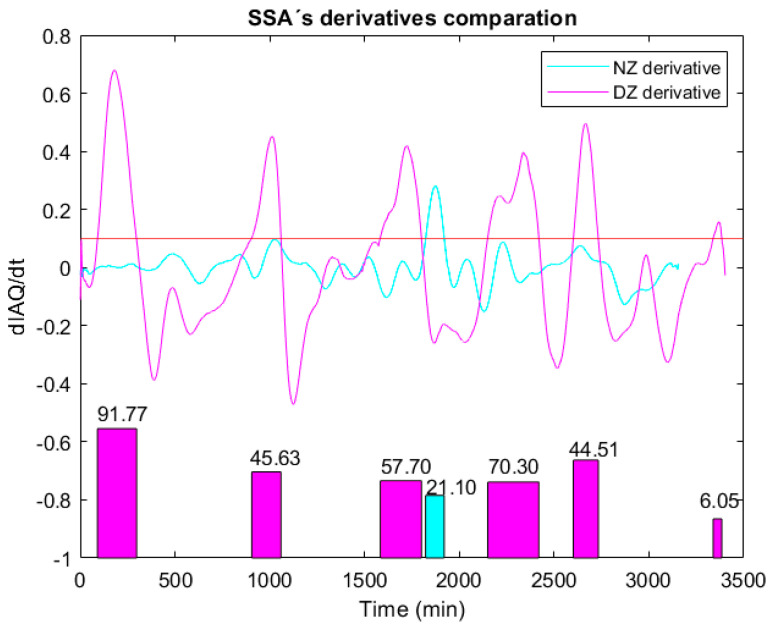
Top: Time derivative of the processed IAQ signal in both cases: without disturbance (NZ) and with disturbance (DZ). Down: Area calculated from the derivative signals with higher level to 0.1%/min; the area value is also referred.

Focused on SSA IAQ time derivative, Figure 15(top) compares the two evaluated scenarios: without (magenta line) and with (blue line) forced disturbance. As can be seen, in the experimental recording performed in the HelpResponder Project, the smoke introduced in the scenario under test generated a time derivative (DZ derivative) with a higher level and duration. In order to better discriminate between the Normal Zone (NZ) and the Disturbance Zone (DZ), the integral was applied to both signals, for |dIAQdt|>0.1%/min values, obtaining the representation shown in Figure 15(down). The interpretation of the area generated from the time derivatives, in both cases, facilitates the detection of air quality anomalies through a threshold for the area value.

## 6. Conclusions

Singular Spectral Analysis is a well-known noisy signals processing technique, that can also be used for time series. It facilitates the study and analysis of a reconstructed signal from its dominant components. In the context of the HelpResponder Project, the authors have highlighted the contribution of SSA to the processing of the air quality signal recorded with commercial sensors at 1 min sampling.

SSA contributes to the modelling and estimation of non-linear time series such as the one addressed in this paper. After a comparative study of Matlab functions for non-linear time series modelling, treepartition was chosen. The estimation result of the IAQ signal processed with SSA, with respect to the original one (raw data), provides a higher fit level, lower MSE error and less computational time. In addition, it facilitates the prediction of future values, such as those shown for air quality with a prediction horizon of one day.

However, where the contribution of SSA stands out most is in the analysis of the time derivative of the series, admitting that the IAQ sensor does not saturate. The derivative of the original signal, with an important high-frequency component, is of little use, as shown in this work. However, after applying SSA, the time derivative of the series provides information of interest, both in terms of reached level (%/s) and time duration. In short, the application of SSA to air quality monitoring offers a different alternative of signal treatment for the rapid detection of degradation, whether due to natural or forced causes (emergencies).

## Figures and Tables

**Table 1:** Interpretation of IAQ based on the Bosch algorithm.

IAQ Index (%) (*)	Air Quality
0–10	Good
10–20	Average
20–30	Little Bad
30–40	Bad
40–60	Worse
60–100	Very Bad

(*) The values are converted to the percentage of the maximum value of 500.

**Table 2:** Results of the validation stage for different nonlinear time series model (order = 50), working with the IAQ original data.

Matlab Function	FIT (%)	MSE	Relative Time
Wavenet	81.67	1.614	2.684
Sigmoidnet	81.77	1.597	15.923
Treepartition	82.24	1.516	1

**Table 3:** Effect of the eigenvalues number on the IAQ time series reconstruction (fit percentage) applying SSA.

SSA: Reconstruction Fit of the IAQ Time Series, *L* = 250					
Eigenvalues	4	6	8	10	12
Fit (%)	80.27	81.33	81.77	82.09	82.42

## Data Availability

Not applicable.

## References

[1] World Health Organization (WHO) (2019). Monitoring Health for the SDGs: Sustainable Development Goals.

[2] Rodríguez-Molano J.I., Obando-Bobadilla L.M., Ruiz-Nieto M.P. (2018). Of cities traditional to smart cities. Proceedings of the 2018 13th Iberian Conference on Information Systems and Technologies (CISTI).

[3] Sanchez L., Galache J.A., Gutierrez V., Hernandez J.M., Bernat J., Gluhak A., Garcia T. SmartSantander: The meeting point between Future Internet research and experimentation and the smart cities. Proceedings of the 2011 Future Network Mobile Summit.

[4] Santos C., Jiménez J.A., Espinosa F. (2019). Effect of event-based sensing on IoT node power efficiency. Case study: Air quality monitoring in smart cities. IEEE Access.

[5] Kalajdjieski J., Stojkoska B.R., Trivodaliev K. IoT Based Framework for Air Pollution Monitoring in Smart Cities. Proceedings of the 28th Telecommunications forum TELFOR 2020.

[6] González E., Casanova-Chafer J., Romero A., Vilanova X., Mitrovics J., Llobet E. (2020). LoRa Sensor Network Development for Air Quality Monitoring or Detecting Gas Leakage Events. Sensors.

[7] Popoola O.A.M., Carruthers D., Lad C., Bright V.B., Mead M.I., Stettler M.E.J., Saell J.R., Jones R.L. (2018). Use of networks of low cost air quality sensors to quantify air quality in urban settings. Atmos. Environ..

[8] Lasomsri P., Yanbuaban P., Kerdpoca O.L., Ouypornkochagorn T. A Development of Low-Cost Devices for Monitoring Indoor Air Quality in a Large-Scale Hospital. Proceedings of the 2018 15th International Conference on Electrical Engineering/Electronics, Computer, Telecommunications and Information Technology.

[9] Polichetti T., Miglietta M.L., Alfano B., Massera E., de Vito S., di Francia G., Faucon A., Saoutie E., Boisseau S., Marchand N. (2019). A Networked Wearable Device for Chemical Multisensing”. Lecture Notes in Electrical Engineering.

[10] Okigbo C.A., Seeam A., Guness S.P., Bellekens X., Bekaroo G., Ramsurrun V. (2020). Low Cost Air Quality Monitoring: Comparing the Energy Consumption of an Arduino against a Raspberry Pi Based System.

[11] Kim J.Y., Chu C.H., Shin S.M. (2014). ISSAQ: An integrated sensing systems for real-time indoor air quality monitoring. IEEE Sens. J..

[12] Sendra S., Garcia-Navas J.L., Romero-Diaz P., Lloret J. (2019). Collaborative LoRa-Based Sensor Network for Pollution Monitoring in Smart Cities. Proceedings of the 2019 Fourth International Conference on Fog and Mobile Edge Computing (FMEC).

[13] Khalifeh A., Darabkh K.A., Khasawneh A.M., Alqaisieh I., Salameh M., AlAbdala A., Alrubaye S., Alassaf A. (2021). Wireless Sensor Networks for Smart Cities: Network Design, Implementation and Performance Evaluation. Electronics.

[14] Lütkepohl H. (2005). New Introduction to Multiple Time Series Analysis.

[15] Brockwell P.J., Davis R.A. (2016). Intoduction to Time Series and Forecasting.

[16] Yongzhi J. (2021). Application of fault detection using distributed sensors in smart cities. Phys. Commun..

[17] Golyandina N.E., Zhigljavsky A. (2013). Singular Spectrum Analysis for Time Series.

[18] Hassani H. (2007). Singular Spectrum Analysis: Methodology and Comparison. J. Data Sci..

[19] Ali-Kazmi S.N., Ulasyar A., Nadeem-Khan M.F. IoT based Energy Efficient Smart Street Lighting Technique with Air Quality Monitoring. Proceedings of the 2020 14th International Conference on Open Source Systems and Technologies (ICOSST).

[20] EPA (2021). Indoor Air Quality|EPA’s Report on the Environment (ROE)|US EPA. https://www.epa.gov/report-environment/indoor-air-quality.

[21] Liu H., Yan G., Duan Z., Chen C. (2021). Intelligent modeling strategies for forecasting air quality time series: A review. Appl. Soft Comput..

[22] Lin Y.-C., Lee S.-J., Ouyang C.-S., Wu C.-H. (2020). Air quality prediction by neuro fuzzy modeling approach. Appl. Soft Comput..

[23] Espinosa R., Palma J., Jiménez F., Kaminska J., Sciavicco G., Lucena-Sánchez E. (2021). A time series forecasting based multi-criteria methodology for air quality prediction. Appl. Soft Comput..

[24] Ljung L. (2020). System Identification Toolbox. User’s Guide.

[25] Ljung L. (2020). System Identification Toolbox. Getting Started Guide.

[26] Ljung L. (2020). System Identification Toolbox. Reference.

[27] Ljung L. (1999). System Identification. Theory for the User.

[28] Marques C.A.F., Ferreira J.A., Rocha A., Castanheira J.M., Melo-Gonçalves P., Vaz N., Dias J.M. (2006). Singular spectrum analysis and forecasting of hydrological time series. Phys. Chem. Earth Parts A/B/C.

